# Successful Treatment of pMMR MSS IVB Colorectal Cancer Using Anti-VEGF and Anti-PD-1 Therapy in Combination of Gut Microbiota Transplantation: A Case Report

**DOI:** 10.7759/cureus.42347

**Published:** 2023-07-24

**Authors:** Xianshuo Cheng, Xiaozheng Li, Xudong Yang, Shaojun Fang, Zhenyu Wang, Tingting Liu, Mengyao Zheng, Maocai Zhai, Zhibin Yang, Tao Shen

**Affiliations:** 1 Colorectal Surgery, Yunnan Tumour Hospital Kunming Medical University No.3 Affiliated Hospital, Kunming, CHN; 2 Life Sciences and Oceanography, Shenzhen University, Shenzhen, CHN; 3 Colorectal Surgery, Kunming Medical University, Kunming, CHN; 4 Microbiology, JCY Biotech Ltd. Pingshan Translational Medicine Center, Shenzhen Bay Laboratory, Shenzhen, CHN; 5 Cardiology, Shenzhen University General Hospital, Shenzhen, CHN

**Keywords:** tumor microenvrionment, immune checkpoint inhibitor, gut microbiota transplantation, pmmr mss crc, metastatic colorectal cancer

## Abstract

Immune checkpoint inhibitors (ICI) have shown great promise in treating advanced or metastatic colorectal cancer (mCRC), especially for CRC patients with deficient mismatch repair (dMMR) and high microsatellite instability (MSI-H). For the remainder of CRC patients presenting with proficient mismatch repair (pMMR) and microsatellite stable (MSS) or low microsatellite instability (MSI-L), ICI showed a low-level response. This study describes a 57-year-old Chinese man diagnosed with pMMR MSS IVb CRC with liver metastasis. Primarily, the patient was administered two consecutive treatments, one composed of an anti-EGFR and modified FOLFOX6 and the other composed of an anti-VEGF and FOLFOXIRI. Due to severe chemotherapy side effects, the patient discontinued treatment and decided to take a third investigational treatment, where an anti-PD-1 and an anti-VEGF were given in combination with fecal microbiota transplantation (FMT) capsules. The patient achieved a partial response (PR), and the tumor size decreased to the extent amenable to surgical resection. After surgery, the patient achieved a pathological complete response (pCR). Patients with pMMR MSS or MSI-L hardly benefit from anti-PD-1 immunotherapy. This study indicated that, to a limited extent, FMT might improve the response to ICI for pMMR MSS CRC patients.

## Introduction

Colorectal cancer (CRC) is the third most common malignancy in the world after breast cancer and lung cancer [1]. In recent years, CRC incidence and mortality rates in China have increased steadily due to changes in lifestyle and diet [2]. In 2020, newly diagnosed CRC cases and CRC-associated deaths in China constituted nearly one-third of all new cases and CRC-related deaths globally, respectively [1]. This has caused a significant burden on the healthcare system. As a result of underdeveloped screening schemes and methodologies, 25% of patients are diagnosed with advanced or metastatic CRC (mCRC) with a 5-year survival rate of about 14% [3]. Chemotherapy and molecular targeted therapy, such as anti-epidermal growth factor receptor (anti-EGFR) and anti-vascular endothelial growth factor (anti-VEGF), have been the first-line treatments for mCRC [4]. However, these treatments only improved survival modestly for heavily treated, advanced refractory CRC patients, and the median survival is about six months [5]. Primary or acquired resistance to these therapies is the main cause of the unsatisfying results. Alternative therapies are in great need for those who progress after first-line treatment.

The discovery and development of immunotherapies have advanced therapeutic approaches against various cancers, such as lung cancer and melanoma. However, many clinical trials and cases have demonstrated that immunotherapy is less effective in CRC. Immunotherapy has shown great clinical efficacy towards untreated deficient mismatch repair (dMMR) and high microsatellite instability (MSI-H) mCRC patients. This patient population accounts for only 15% of the total CRC population [6]. For the rest of the patients with proficient mismatch repair (pMMR) and microsatellite stable (MSS) mCRC, the use of immunotherapy such as anti-PD-1/PD-L1 has not conferred major clinical benefit [7].

Fecal microbiota transplantation (FMT) is a transplantation of gut microbiota from healthy donor to patient, which helps rebuild the balance of patients' gastrointestinal microbial environment. FMT has become an emerging biotherapeutic owing to the increasing understanding of the relationship between disordered gut microbiota consortia and many diseases [8]. It has been proven to be effective in treating many gastrointestinal disorders, such as inflammatory bowel disease (IBD) and Clostridium difficile infection (CDI) [9-10]. Recently, the therapeutic effects of FMT on immune response have been noted. It has been found that metabolites of gut bacteria act directly on intestinal mucosal immune cells.

An example is their ability to activate T cells, dendritic cells, and macrophages [11]. Recently, some cases have been reported in which the control of intestinal microorganisms through FMT affects the immune system and improves immune-mediated diseases. In 2021, FMT was reported to positively influence the response of tumors to anti-PD1 in patients with anti-PD-1 refractory metastatic melanoma in a Phase I clinical trial [12-13]. Researchers found that oral application of a mixture of Clostridiales bacteria alone successfully treated CRC independently of anti-PD-1 in animal models [14]. To date, no clinical trials have been conducted regarding the efficacy of FMT on the response of tumors to anti-PD-1 therapy in pMMR MSS or low microsatellite instability (MSI-L) mCRC patients.

## Case presentation

**Figure 1:**
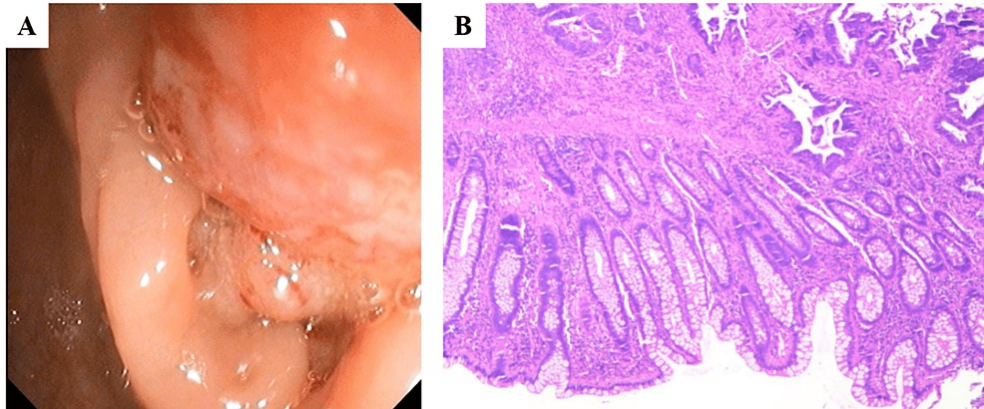
Colonoscopy and histopathology of colon and colon tissue. A. Colonoscopy showing a nodular cauliflower-like tubercle 70cm from the anus verge. B. Biospy of the colon tissue sample showing signs of adenocarcinoma.

**Figure 2:**
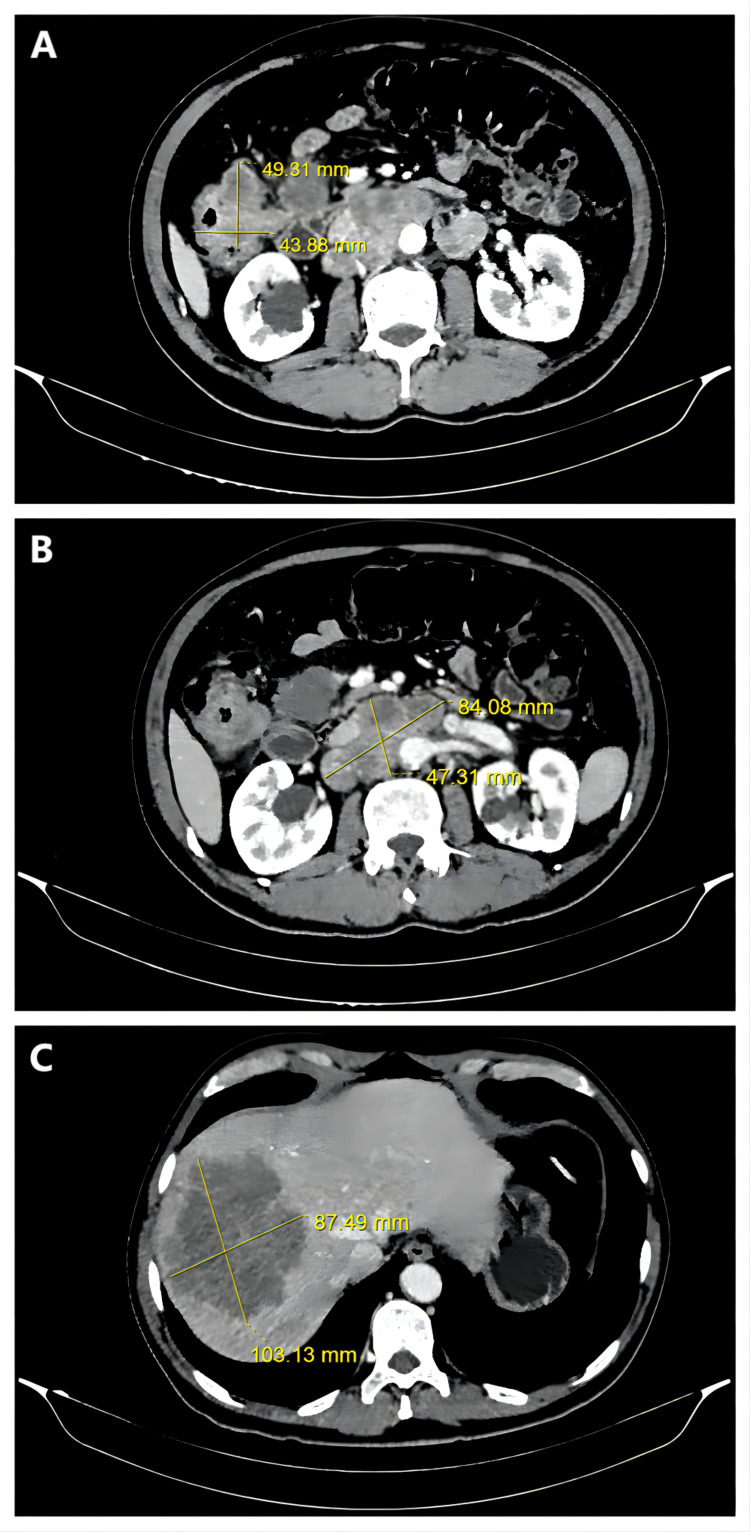
Axial CT images of the patient's abdomen on hospitalization illustrates the sizes of the lesions. A. Colon tumor; B. Lesion of the lymph node metastases; C. Liver metastasis; CT: Computed Tomography.

A 57-year-old Chinese man diagnosed with pMMR cT4bN2M1b IVb mCRC at ascending colon with secondary liver malignancy was hospitalized. The patient had suffered from recurrent abdominal pain with abnormalities in stool shapes for half a month. The tumor marker levels were 30.1 μg/L and 62.64 kU/L for carcinoembryonic antigen (CEA) and carbohydrate antigen 19-9 (CA19-9), respectively (Table 1). A colonoscopy revealed a nodular cauliflower-shaped tubercle 70cm from the anus verge (Fig 1A). A tissue sample biopsy showed features consistent with adenocarcinoma (Fig 1B). Computed Tomography (CT) on the colon showed that the intestinal wall of ascending colon near the hepatic flexure significantly thickened with mass formation. The tumor size was about 4.9cm×4.4cm (Fig 2A) (the tumor sizes reported in this article were the largest cross-section area of the tumors). Multiple enlarged lymph nodes can be seen around the diseased intestinal canal, in the upper, middle, and lower abdominal cavity and retroperitoneum. The size of the large lesion of retroperitoneal lymph node metastases was about 8.4cm×4.7cm (Fig 2B). CT of the liver showed multiple patchy, nodular, low-density shadows, with the larger one on the right lobe of the liver. The size of the tumor on the right lobe of the liver was about 10.3cm×8.7cm (Fig. 2C). The KRAS/NRAS/BRAF genetic mutation test, showed that the patient belonged to KRAS/NRAS/BRAF wild type.

**Table 1:** Tumor marker levels after different stages of treatments. CEA: Carcinoembryonic Antigen. CA19-9: Carbohydrate Antigen 19-9. The reference value of CEA for healthy, non-smoking adults is less than 3 μg/L. The reference value of CA19-9 for healthy adults is less than 37 kU/L.

	CEA	CA199
On hospitalization	30.1 ug/L	62.64KU/L
After first treatment	20.9 ug/L	50.18 KU/L
After second treatment	9.31 ug/L	50.14 KU/L
After third treatment	5.38 ug/L	30.61 KU/L

First treatment

**Figure 3:**
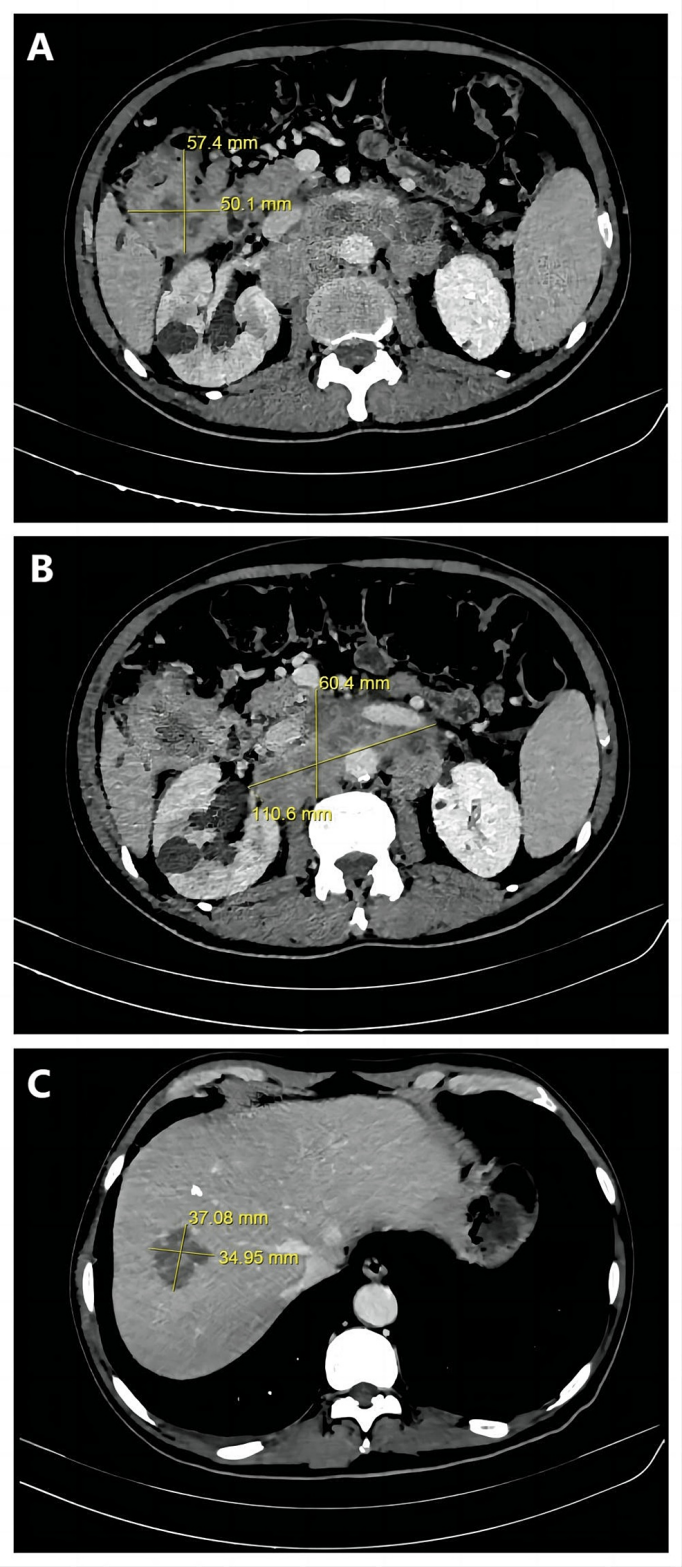
Axial CT images of the abdomen of the patient after the first treatment illustrating the sizes of the lesions. A. Colon tumor. B. Lesion of the lymph node metastases. C. Liver metastasis. CT images showed that the liver metastasis reduced while the colon tumor and lymph node metastases grew in size. Progressive disease (PD) was achieved after the first treatment. CT: Computed Tomography.

The patient was first enrolled in Phase II clinical trial to assess the efficacy and safety of CPGJ602, a monoclonal antibody, in combination with chemotherapy for the treatment of KRAS/NRAS/BRAF wild type mCRC (Plan No. SSGJ-602-mCRC-II-01). The patient received CPGJ602 [540mg by intravenous guttae on the first day of every treatment cycle (540mg get d1)], plus mFOLFOX6 [Oxaliplatin (L-OHP) 140mg/m^2^ ivgtt d1, fluorouracil (5-FU) 4025mg/m^2^ by micropump 44-48h, 5-FU 675mg/m^2^ by intravenous injection (iv) d1, cisplatin, and fluorouracil (CF) 670mg/m^2^ ivgtt d1] biweekly from 2021/09/17 to 2021/11/12 for a total of 5 cycles. CPGJ602 is a recombinant anti-EGFR human-mouse chimeric monoclonal antibody. After 5 cycles of therapies, CT showed liver metastasis on the right lobe reduced from 10.3cm×8.7cm to 3.7cm×3.5cm (Fig 3C). However, the colon tumor grew from 4.9cm×4.4cm to 5.7cm×5.0cm (Fig 3A). The lesion of the retroperitoneal lymph node metastases grew from 8.4cm×4.7cm to 11.1cm×6.0cm (Fig 3B). The tumor markers were CEA: 20.90 μg/L and CA19-9: 50.18 kU/L (Table 1). After comprehensive radiology and multidisciplinary team (MDT) assessment, the patient's disease was confirmed to have progressed to yc T4bN2M1c IVc mCRC.

Second treatment

**Figure 4:**
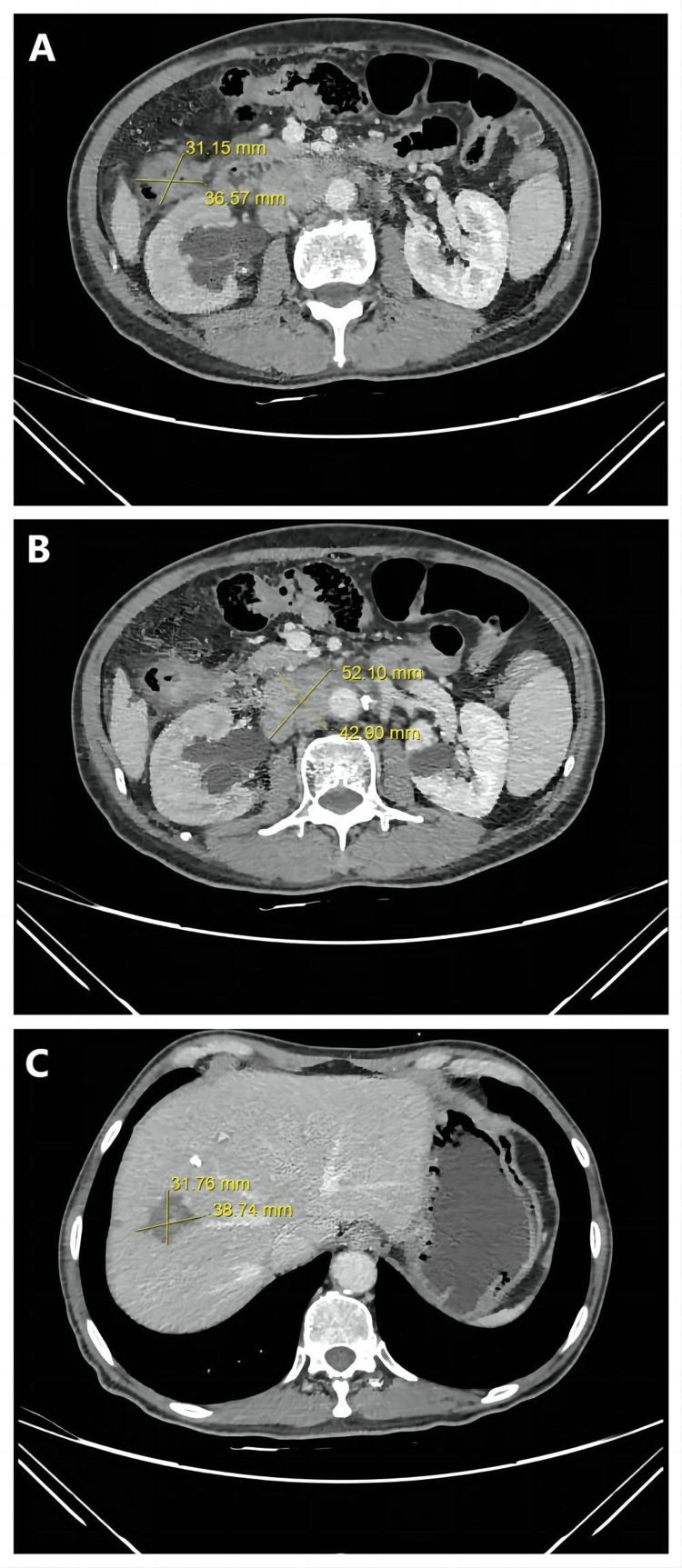
Axial CT images of the abdomen of the patient after the second treatment illustrating the sizes of the lesions. A. Colon tumor. B. Lesion of the lymph node metastases. C. Liver metastasis. The sizes of the colon tumor and lymph node metastases reduced while size of liver metastasis remained relatively unchanged after the second treatment. PR (partial response) was achieved.  CT: Computed Tomography.

A second treatment was given following the failure of the previous one. The therapy consisted of Bevacizumab (290mg ivgtt d1) plus FOLFOXIRI [L-OHP 130mg/m2 ivgtt d1, 5-FU 450mg/m^2^ micropump 44-48h, irinotecan (CPT) 260mg/m^2^ ivgtt d1, leucovorin (LV) 600mg/m^2^ ivgtt d1] biweekly from 2021/11/25 to 2021/12/25 for total of three cycles. CT results showed that partial response (PR) was achieved after the treatment. The size of the colon tumor reduced from 5.7cm×5.0cm to 3.6cm×5.0cm, and the size of the retroperitoneal lymph node metastases reduced from 11.1cm×6.0cm to 6.2cm×3.6cm. The size of the tumor on the liver remained relatively unchanged from 3.7cm×3.5cm to 3.8cm×3.0cm. Tumour marker testing showed CEA with a value of 12.5 μg/L and CA19-9 with a value of 47.75 kU/L (Table 1). The treatment was continued for another three cycles from 2021/12/25 to 2022/02/8. The size of the colon tumor, retroperitoneal lymph node metastases, and liver metastasis remained relatively unchanged from 3.6cm×5.0cm to 3.7cm×3.1cm (Fig 4A), from 6.2cm×3.6cm to 5.2cm×4.3cm (Fig 4B), and from 3.8cm×3.0cm to 3.9cm×3.2cm (Fig 4C), respectively. Tumour marker testing showed CEA with a value of 9.31 μg/L and CA19-9 at 50.14 kU/L (Table 1).

Third treatment

**Figure 5:**
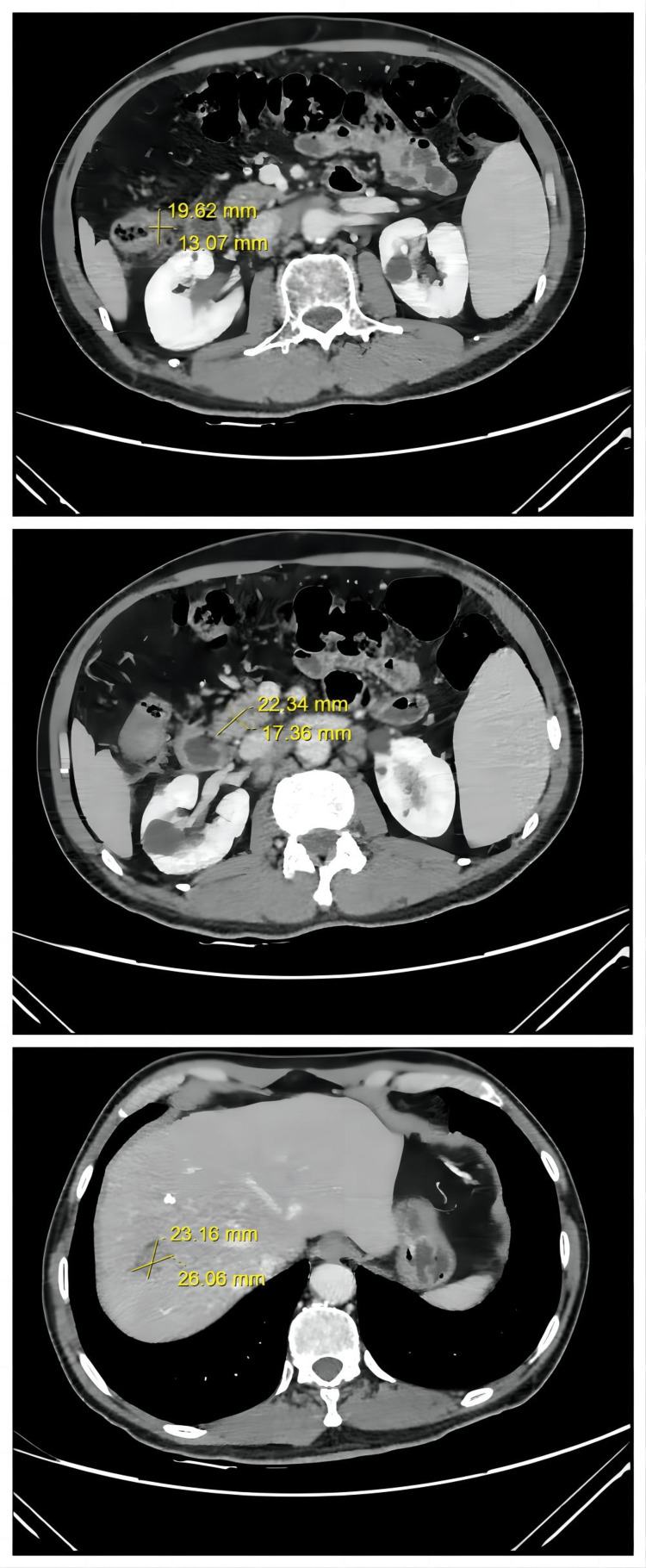
Axial CT images of the patient's abdomen after the third treatment illustrate the sizes of the lesions. A. Colon tumor. B. Lesion of the lymph node metastases. C. Liver metastasis. After the third treatment, the sizes of the colon tumor, lymph node metastases, and liver metastasis all reduced. PR (partial response) was achieved. CT: Computed Tomography.

After the second treatment, side effects, i.e., digestive tract reaction and bone marrow suppression caused by high-intensity chemotherapy, made the patient unwilling to continue the treatment. The condition of the patient did not allow curative surgical resection. The patient was then enrolled into a new investigative treatment plan where Tislelizumab (200mg ivgtt d7), an anti-PD1 ICI from BeiGene, was used in combination with Bevacizumab (270mg ivgtt d7) after oral ingestion of gut microbiota capsules. The treatment was given every three weeks from 2022/03/02 to 2022/07/01 for six cycles. In each cycle, one gut microbiota capsule provided by Shenzhen JCY Biotech Co., Ltd. was given orally daily for the first four days. Stool samples were taken at the start of treatment cycles 1, 5, and 6 for metagenomic analysis. On the seventh day, Tislelizumab and Bevacizumab were given intravenously. CT results showed that PR was achieved after the treatment. The size of the tumor on the colon reduced from 3.7cm×3.1cm to 2.1cm×1.7cm, the size of the retroperitoneal lymph node metastases reduced from 5.2cm×4.3cm to 2.3cm×1.4cm and the size of tumor on the liver reduced from 3.9cm×3.2cm to 2.9cm×2.3cm. Tumour marker testing showed CEA with a value of 4.3 μg/L and CA19-9 with a value of 23.97 kU/L (Table 1). The treatment then continued for another 2 cycles from 2022/07/01 to 2022/08/23. Stool samples were taken at the start of treatment cycles 7 and 8 for metagenomic analysis. CT results showed stable disease (SD) after the 2 treatment cycles. The size of tumors on the colon, lymph node, and liver remained relatively unchanged from 2.1cm×1.7cm to 2.0cm×1.3cm (Fig 5A), from 2.3cm×1.4cm to 2.2cm×1.7cm (Fig 5B), and from 2.9cm×2.3cm to 2.6cm×2.3cm, respectively (Fig 5C). Tumour marker testing showed CEA with a value of 5.38 μg/L and CA19-9 with 30.61 kU/L (Table 1).

**Figure 6:**
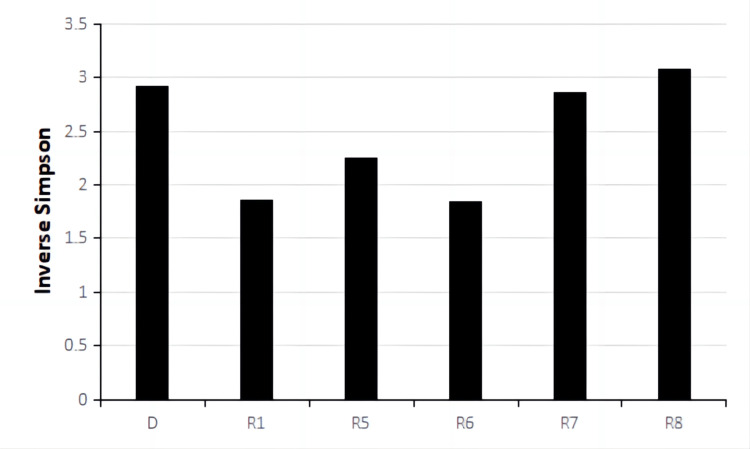
Inverse Simpson Alpha Diversity of fecal microbiota samples from the donor and patient during the third treatment. D represented the sample from the donor. R1, R5, R6, R7, and R8 represented samples from the patient taken before treatment cycle 1, treatment cycle 5, treatment cycle 6, treatment cycle 7, and treatment cycle 8 during the third treatment.

**Figure 7:**
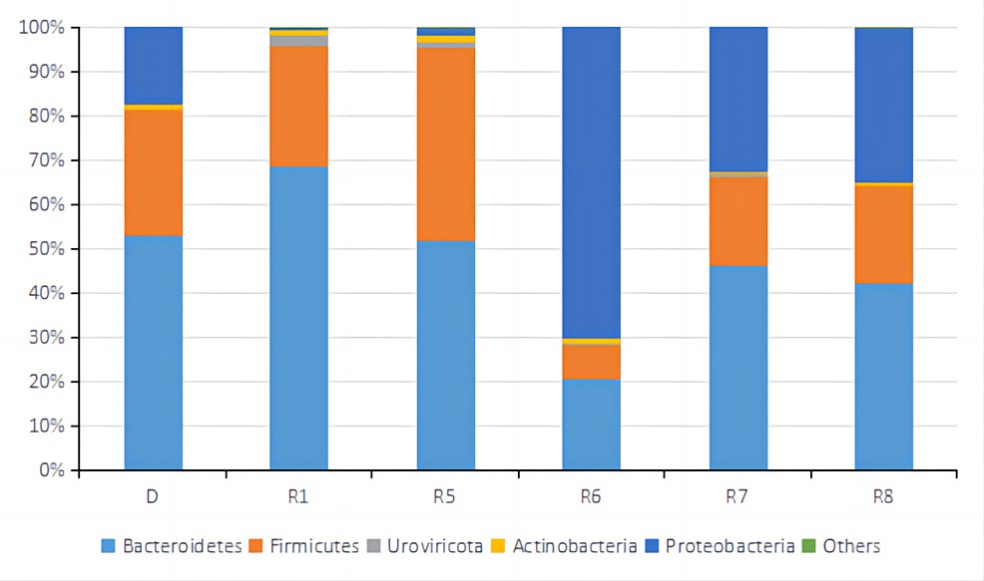
Relative abundance of top 5 OTUs at the Phylum level of fecal microbiota samples from the donor and patient during the third treatment. D represented the sample from a donor. R1, R5, R6, R7, and R8 represented samples from the patient taken before treatment cycle 1, treatment cycle 5, treatment cycle 6, treatment cycle 7, and treatment cycle 8 during the third treatment. OUTs: Operational Taxonomic Units.

**Figure 8:**
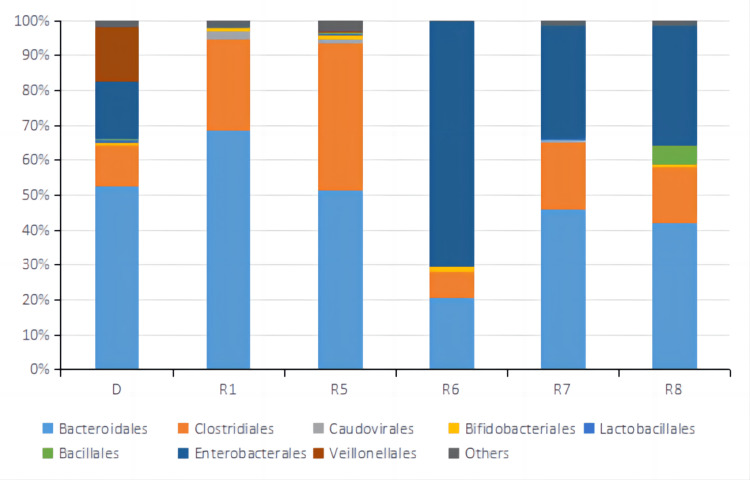
Relative abundance of top 8 OTUs at the Order level of fecal microbiota samples from the donor and patient during the third treatment. D represented the sample from a donor. R1, R5, R6, R7, and R8 represented samples from the patient taken before treatment cycle 1, treatment cycle 5, treatment cycle 6, treatment cycle 7, and treatment cycle 8 during the third treatment. OUTs: Operational Taxonomic Units.

**Figure 9:**
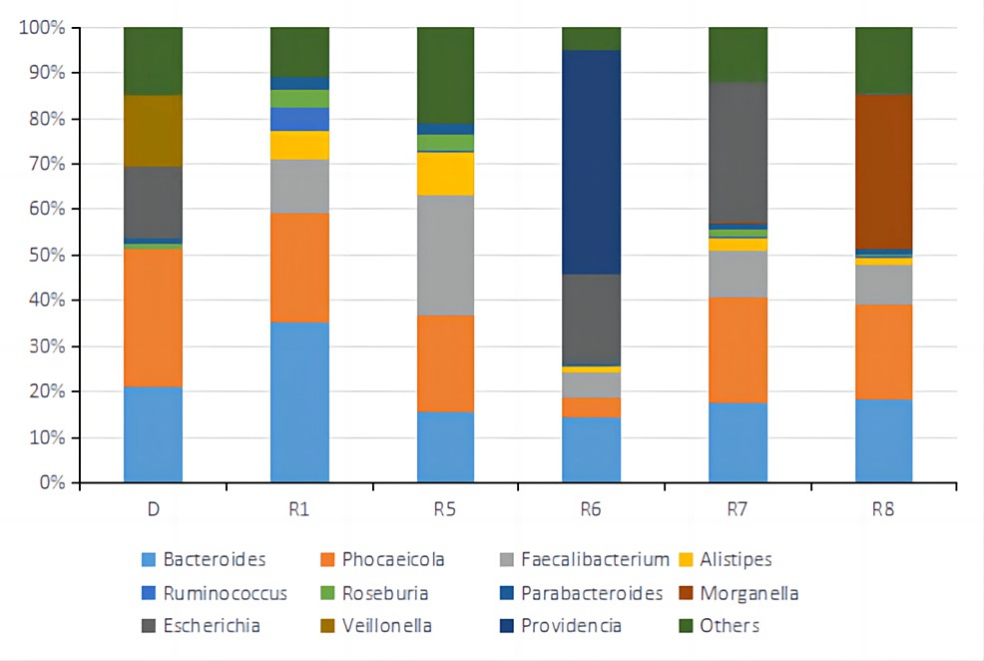
Relative abundance of top 11 OTUs at the Genus level of fecal microbiota samples from the donor and patient during the third treatment. D represented the sample from a donor. R1, R5, R6, R7, and R8 represented samples from the patient taken before treatment cycle 1, treatment cycle 5, treatment cycle 6, treatment cycle 7, and treatment cycle 8 during the third treatment. OUTs: Operational Taxonomic Units.

**Figure 10:**
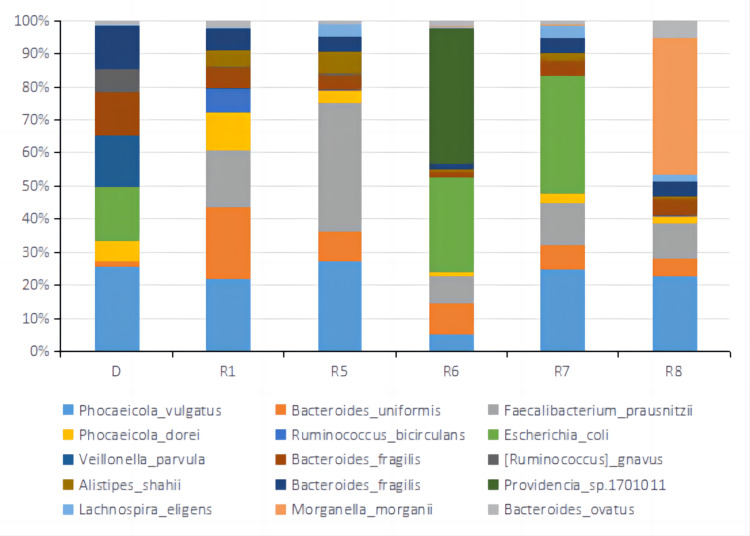
Relative abundance of top 15 OTUs at the Species level of fecal microbiota samples from the donor and patient during the third treatment. D represented the sample from a donor. R1, R5, R6, R7, and R8 represented samples from the patient taken before treatment cycle 1, treatment cycle 5, treatment cycle 6, treatment cycle 7, and treatment cycle 8 during the third treatment. OUTs: Operational Taxonomic Units.

After eight treatment cycles, MDT discussion concluded that the lesions on the colon and liver significantly decreased, and some became invisible on radiological images. PR was achieved, and curative surgical resection could be performed to remove the lesions. Microwave ablation (MWA) was subsequently conducted for liver metastasis and open radical right hemicolectomy for the colorectal primary tumor and associated lymph nodes. Liver metastases located near the diaphragmatic surface of the right lobe of the liver were removed by ablation, and the liver was negative for malignancy by histopathology. Postoperative histopathological examination of the colon and lymph node revealed acute and chronic inflammation with local low-grade intraepithelial neoplasia of glandular epithelium, and no cancer tissues were observed. The patient achieved a pathological complete response (pCR). No severe side effects were observed. The summary of tumor sizes and marker levels during all treatments are shown in Tables 1, 2. The alpha diversity of the gut microbiota was shown in Fig 6 based on Inverse Simpson. The relative abundance of top operational taxonomic units (OTUs) at Phylum, Order, Genus, and Species levels are shown in Fig 7-10.

**Table 2:** Sizes of the colon tumor, lesion of retroperitoneal lymph node metastases, and liver metastasis after different stages of treatments. CT: Computed Tomography.

	Colon tumor (CT)	Lymph node metastases （CT）	Liver metastasis （CT）
On hospitalization	4.9cm×4.4cm	8.4cm×4.7cm	10.3cm×8.7cm
After first treatment	5.7 cm×5.0cm	11.1cm×6.0cm	3.7cm×3.5cm
After second treatment	3.7cm×3.1cm	5.2cm×4.3cm	3.9cm×3.2cm
After third treatment	2.0cm×1.3cm	2.2cm×1.7 cm	2.6cm×2.3cm

## Discussion

CRC is a major type of cancer-causing the second-largest number of deaths in the world. Early diagnosis of CRC through regular screening has significantly improved the 5-year survival of patients. About 25% of patients are still diagnosed with stage 4 disease, and up to half of the early-stage patients progress to mCRC [15]. The prognosis of mCRC patients is poor, with a 5-year median survival rate of 14% only in the USA [3].

CRC can be categorized into tumors that are dMMR-MSI-H (15%) with a high overall mutation burden and pMMR-MSI-L or pMMR-MSS (85%) with a much lower mutation burden [16]. For mCRC, the proportion of dMMR-MSI-H further decreases to 5% [17]. In recent years, ICI had a long record of achieving long-term responses in previously refractory solid tumors such as lung cancer and melanoma. In 2017, ICI received regulatory approval for dMMR-MSI-H CRC [18]. However, ICI has proved ineffective for most CRC patients who are pMMR-MSI-L or pMMR-MSS. In the Phase II clinical trial, the immune-related objective response rate was 0 for pMMR CRC patients receiving pembrolizumab [19].

Substantial efforts have been taken to address the resistance to ICI. Among them, the modulation of gut microbiota is very promising [20], which has been shown to have a heavy impact on the development and function of the immune system [21]. A phase I clinical trial assessed the safety and feasibility of FMT in combination with anti-PD-1 against metastatic melanoma in ten previously treated anti-PD-1-refractory patients [12]. Out of ten patients, clinical responses were observed in three, which included two PR and one CR. The effect was associated with favorable changes in immune cell infiltrates and gene expression profiles in the tumor microenvironment and gut lamina propria.

A few studies suggested that CRC patients refractory to ICI could benefit from gut microbiota modulation [22-23]. Clinical trials examining the exact efficacy of FMT in treating pMMR CRC together with ICI are still ongoing (NCT04729322 and NCT05279677). In our case, we demonstrated that microbiota modulation could, to an extent, activate the response of tumors to ICI for a pMMR-MSI-L mCRC patient. FMT was combined with Tislelizumab and Bevacizumab in treating pMMR MSI-L mCRC patients after two first-line treatments. In each treatment cycle, gut microbiota capsules were taken orally on days 1-4 to modulate the microbial environment in the patient's gut. Tislelizumab and Bevacizumab were injected on day seven. Without chemotherapy, the sizes of colon tumors, lymph node metastases, and liver metastasis decreased significantly, as did the tumor makers. Curative resection was performed to achieve pCR.

Metagenomic sequencing was performed on stool samples before treatment cycles 1, 5, 6, 7, and 8. Metagenomic sequencing is a powerful technique used to analyze the genetic material extracted from a complex microbial community, such as the gut microbiota. It provides a comprehensive view of the community's microbial composition and functional potential. Alpha diversity based on Inverse Simpson Index was calculated for each sample shown in Fig 6. Inverse Simpson Index, also known as the Simpson's Diversity Index, is a metric used to quantify the diversity or richness of a microbial community. It considers the number of different microbial species (richness) and their relative abundance (evenness). A higher Inverse Simpson Index indicates a more diverse and balanced microbial community. Microbiome diversity gradually increased from 1.86 (R1) to 3.10 (R8), similar to the donor's level of 2.92 (D), suggesting a beneficial effect of FMT on the gut microbial environment. In recent studies, the high diversity of gut microbiota correlates closely with anti-PD-1 immunotherapy responses [12,20,24,25]. Looking at the relative abundance of top OTUs at Phylum, Order, Genus, and Species level (Fig 7-10), there was a significant discrepancy between D and R1, suggesting that microbiota compositions of the donor and the patient were greatly different. From R1 to R5, a favorable transition of microbiota composition was observed. The proportions of Phylum Bacteroidetes that did not favor anti-PD-1 response decreased while Phylum Firmicutes that favored anti-PD-1 response increased (Fig 7) [20].

Similar trends could be seen at Order and Genus levels (Fig 8, 9). Communities that favored PD-1 response, such as Order Clostridiales and Genus Faecalibacterium, increased in percentage, while communities that did not favor PD-1 response, such as Order Bacteroidales and Genus Bacteroides, decreased relative numbers. At the level of Species, it was noted that the relative abundance of Faecalibacterium prausnitzii, which was reported to enrich in ICI responders' stool samples [20,26-27], increased significantly from R1 of 12% to R5 of 26%. Bacteroides uniforms, which was reported to enrich in ICI non-responders' stool samples [13], decreased from R1 of 15% to R5 of 6.2% in relative number (Fig 10). It was evident that FMT modulated the gut microbial microbiota of the patient in a favorable way toward IC therapy response. The tumor sizes were reported to reduce significantly between treatment cycle 1 and treatment cycle 6, suggesting a possible correlation between a favorable gut microbiota environment and anti-PD-1 therapy efficacy. From R6 to R8, the microbiota composition shifted dramatically to an unfavorable condition, which might lead to the SD result for treatment cycles 7 and 8. By comparing the relative abundance and distribution of fecal microbiota, researchers and clinicians can gain insights into the potential therapeutic effects of FMT and identify any shifts in the microbial community associated with treatment outcomes.

## Conclusions

The report described the first case to treat a Stage IV pMMR MSS mCRC patient using Tislelizumab and Bevacizumab in combination with FMT capsules. The investigational therapy achieved pCR by reducing the tumor size to the range feasible for curative surgery. pMMR MSS CRC was reported to respond to immunotherapy hardly. The case showed that FMT has great potential in immunotherapy for patients with pMMR MSS CRC to improve response to ICI and patients' survival.

## Appendices

CPGJ602 is a recombinant anti-EGFR human-mouse chimeric monoclonal antibody developed by  Sunshine Guojian Pharmaceutical (Shanghai) Co., Ltd. CPGJ602 plus mFOLFOX6 against KRAS/NRAS/BRAF wild-type mCRC was in phase II clinical trial under Plan No. SSGJ-602-mCRC-II-01. The detail of Plan No. SSGJ-602-mCRC-II-01 can be found on the American Society of Clinical Oncology website under Abstract #:3574 at https://meetings.asco.org/abstracts-presentations/208505.  Tislelizumab is a humanized IgG4 anti-PD-1 monoclonal antibody developed by BeiGene. Tislelizumab was approved by China's National Medical Products Administration (cFDA). More information can be found at https://www.beigene.com/our-science-and-medicines/tislelizumab/.
